# How does the supervision effect affect the firm’s performance in Taiwanese stock market?

**DOI:** 10.1371/journal.pone.0307988

**Published:** 2024-09-05

**Authors:** Tsung-Yu Hsieh, Tsai-Yin Lin, Fangjhy Li, Chih-Yun Tien

**Affiliations:** 1 Department of Economics, Economics and Management College, Zhaoqing University, Zhaoqing, Guangdong, P. R. of China; 2 Department of Finance, National Kaohsiung University of Science and Technology, Kaohsiung City, Taiwan; 3 Department of Finance, School of Finance, Hubei University of Economics, Wuhan, Hubei Province, P.R. China; University of Baltistan, PAKISTAN

## Abstract

This paper explores the impact of the CEO’s supervisory role as an independent director on firm performance, utilizing measures such as Return on Equity (ROE) and Tobin’s Q. The supervisory effect is assessed through the remuneration of directors and supervisors. Empirical findings indicate a significant supervisory role when the CEO also serves as an independent director. Whether viewed from the perspective of shareholders’ equity or company growth, the CEO’s involvement as an independent director positively influences firm performance. Furthermore, the concurrent roles of CEO and independent director contribute to enhanced firm performance, irrespective of their relationship.

## Section 1: Introduction

The board of supervisors provides supervision, and independent directors provide decision-making supervision [1]. Based on the capital market, this study examines the influence of private enterprise owners on the independence of supervisory boards. Using the data of Chinese listed companies, the empirical results show that the regulatory agencies of Chinese listed companies are not only inefficient but also lack independence [2]. With the rapid development of the economy and the continuous expansion of the company’s scale, under the condition of separation of ownership and management rights, the overall salary cost of employees has a significant impact on the future development of the company. The issue of CEO compensation has also become a topic of concern to shareholders. The paper [3] points out that due to the separation of ownership and management rights, the interests of management and shareholders depend on the monitoring mechanism, resulting in agency problems. A large body of financial literature supports the agency theory that centralized board membership will provide better oversight of corporate governance [4–6], as decentralized boards lead to communication barriers and reduce board effectiveness. An ineffective board of directors fails to exercise proper oversight of the company, thereby affecting agency problems between management and shareholders.

Since 2002, Taiwan’s competent authorities have introduced the independent director system from the OTC listing rules to enhance national competitiveness, reduce financing costs, and conform to international trends. In recent years, economic uncertainty has been heightened, including as a result of economic shocks, e.g., Covid-19. In 2006, the law revised the independent director system in the Securities Exchange Law. Article 14–2 of the Securities Exchange Law, it is clearly stated that independent directors are also directors. However, compared with ordinary directors, independent directors have qualification restrictions, mainly due to the gender, professionalism, and limitations of independent directors. In 2017, the establishment of independent directors was fully regulated.

The purpose of this work is to examine the supervisory effect of the CEO concurrently serving as an independent director and the firm’s performance from 2009 to 2019. Firm performance is measured by ROE and Tobin’s Q, respectively and directors’ compensation of supervisors measures the supervisory effect. The contributions of this work are as follows:

First, this paper examines the relationship between independent directors and changes in CEO compensation. Compensation is one of the deciding factors for accepting outside directors. There is a positive correlation between the firm’s performance and the changes in the remuneration of directors and supervisors. However, in the previous literature, there was no direct discussion of the CEO serving as an independent director; both the CEO and the independent director were regarded as leaders of the same company [7], and some scholars found that independent directors have a very important role in the board of directors. The effectiveness of oversight is influenced by the board of directors. As the size of the company expands, both the size of the board of directors and the number of independent directors increases accordingly.

Second, this paper examines the supervisory role of the CEO as an independent director from three perspectives. Where, shareholders’ equity, company growth, and directors’ and supervisors’ remuneration are used as proxy variables. The executive power of the CEO with independent directors will have a better supervisory effect than the CEO who does not concurrently serve as independent directors. In other words, whether the increase in the remuneration of directors and supervisors lead to the improvement of the firm’s performance?

This paper considers whether the CEO’s concurrent appointment as an independent director of other companies may affect his supervisory effect. Generally, remuneration is one of the determining factors that influence whether to accept the role of outside director [8]. This paper finds that the role of the CEO as an independent director has a higher supervisory role. From the perspective of shareholders’ equity or company growth, the CEO concurrently serving as an independent director will improve the firm’s performance.

The remainder of this paper is organized as follows. Section 2 discusses the literature review and hypothesis development, and Section 3 describes data sources and variable measurements. Section 4 obtains empirical results. Finally, Section 5 presents the conclusions and final discussion.

## Section 2: Literature review and hypothesis development

The board of directors plays an important role in the formation of a firm’s organization, improving corporate management and business performance by supervising, consulting, and utilizing external resources. The board plays a role in providing business strategy and oversight services to facilitate financing management. Operation is to provide board experience and professional competence; supervision is to ensure the growth of the company and the interests of shareholders. Through the supervision function, the board of directors coordinates the interests of managers and shareholders, thereby reducing agency costs and protecting the interests of shareholders. When the enterprise develops and grows, it will issue shares to the public to facilitate financing, so that the proportion of external shares will gradually realize the separation of ownership and management rights. The CEO compensation contract is seen as an important mechanism to solve agency problems within the firms and helps to align shareholder and management goals [9]. Empirically, This paper [10] found that the relationship between managers’ compensation and performance was not significant based on their working experience, inconsistent with the proposition of this paper [9]. Both the CEO and independent directors are regarded as the leaders of peer companies [7]; even some scholars have found that the influence of independent directors is higher than the influence of the board of directors. This paper [11] even found that after the death of independent directors, the company’s stock price fell sharply, implying that they may play an important role in corporate governance.

Boards dominated by outsiders are more likely to eliminate the performance of CEOs [12]. Shareholder wealth increases as outsiders join boards. Therefore, it can be expected that the increase of outsiders on the board of directors will help the monitoring effect. This paper considers the CEO with decision-making power in management. Independent directors served as CEOs should better perform their supervisory duties. Both CEOs and independent directors are viewed as leaders of peer firms [7], and independent directors influence boards. The assumptions are as follows:

**Hypothesis 1**: Firm performance is related to whether the CEO concurrently serves as an independent director.

Other literature also shows that board decisions tend to greatly affect CEO compensation. Directors and supervisors have always been regarded as an important mechanism for supervising managers and one of the factors of corporate governance. The company also pays high remuneration to directors and supervisors. The remuneration of directors and supervisors reflects the supervisory role of the company. Compensation is often one of the deciding factors for taking over as an outside director. The higher the shareholding ratio of outside directors, the stronger their willingness to supervise senior managers, and the lower the possibility of corporate fraud. Therefore, outside directors do have a supervisory role over the company [7, 13, 14]. If the CEO and independent directors are in the relevant industry chain, they will have more decision-making power and supervision capabilities. The assumptions are as follows:

**Hypothesis 2**: The supervision effect of CEOs in the same industry is not the same as that of CEOs in different industries.

## Section 3: Data sources and variable measurements

This paper intends to explore whether a CEO who is also an independent director has executive power (CEO) and the ability to supervise the company (independent director) and whether the effectiveness of supervision will be affected by factors such as business needs. To diversify board members and strengthen board functions, Taiwan’s competent authorities have introduced and promoted the independent director system, stipulating that all listed companies should be equipped with independent directors. Data are taken from the Taiwan Economic Journal Database (TEJ), excluding firms with missing or incomplete information for the year, and firms belonging to the financial industry (different reporting standards). According to the above criteria, the final sample period is from 2009 to 2019, with an average of 726 CEOs serving as independent directors each year. A total of 7,950 listed companies participated in this work during the study period. The corporate governance variables used in this work refer to the past literature on concurrent directors. Corporate financial soundness depends on financial ratio, which can enhance a company’s competitive position and eliminate potential financial risks [15]. All data are in the manuscript and/or supporting information files. See Table 1 for definitions and abbreviations.

**Table 1:** Definitions of explanatory variables.

Abbreviations	Definitions
Independent director supervision effect variable	
1.Chair	1 for independent directors, 0 for non-independent directors
2.Chair_ratio	number of CEOs and independent directors/total number of board seats
3.Num_Chair	Number of CEOs and independent directors of a single listed company
4.Ind_industry	if it belongs to the same industry, set it to 1; otherwise, 0
5.Ind_ChainIndustry	If relevant is set to 1, non-related is set to 0
6.Ind_Tse	Whether the CEO company is a listed OTC company
Corporate Governance Variables	
7.Size	ln (market value)
8.Age	The company’s establishment year to the company’s current year
9.Dir	Number of seats on the board of directors
10.Ind_Dir	Number of independent board seats
11.Ind_ratio	Number of Independent Director seats/ Total Number of Director seats
12.Education	0 for nondisclosure; 1 for high school and below;2 for university;3 for master’s degree; 4 for PHD
13. Institutional_ratio	Total Foreign Shareholding / Number of Outstanding Shares
14. Dir_ownership	Number of Shares held by Directors /Number of Outstanding Shares of the Company × 100
15.Manager_ownership	Number of Shares held by Managers / Number of Outstanding Shares of the Company × 100
Performance Variable	
16.ROE	Net Profit after Tax / Total Shareholders’ Equity
17.Tobin’s Q	(Company Market Value + Book Value of Total Liabilities) / Book Value of Total Assets
Other Control Variables	
18. Duality	Whether the chairman is also the general manager
19. Dir_manager	Number of director and manager seat
20. Dir_manager_ratio	Total Salary of Directors and Supervisors / Number of Salary of Directors and Supervisors
21. MeanSalary_Dir	(Total Salary Manager / General Manager)/Vice Total Salary
22. DUMindustry	The current industry is 1; otherwise, 0
23. DUMyear	The current year is 1; otherwise, 0

## Section 4: Empirical results

### Summary results

The summary results show that the comprehensive quality of independent directors has been significantly improved. This paper finds that the CEO also serves as an independent director to participate in decision-making, which has a high supervisory role. During the study period, the high-tech and biotech industries had more CEO and independent director appointments. The following 4 companies have been awarded, namely Tsan Kun (code = “2430”) in 2011, Xu Fu (code = “4119”) in 2014, Kun Ying (code = “2365”) in 2017, Kun Ying (code = “2365”) in 2018 respectively belong to biotechnology and medical, electronic channel industry, computer and peripheral equipment. During the study periods, there are more CEOs and independent directors in high-tech industries and biotechnology industries. Results are omitted for brevity but are available upon request.

Table 2 lists the Pearson correlation coefficient and Spearman correlation coefficient between variables. It can be found from panel A that corporate governance variables are highly correlated, which is consistent with previous literature. The year of company establishment is only positively correlated with the number of boards of directors, indicating that the longer the company has been established, the larger the size of the board of directors, and it is significantly negatively correlated with other corporate governance variables.

**Table 2:** Correlation analysis (Pearson’s on the upper right/ Spearmen on the lower left).

	01	02	03	04	05	06	07	08	09	10
Panel A: Measuring Corporate Governance Variables										
01 Size		0.008	-0.009	0.150***	0.139***	0.073***	0.269***	-0.021*	-0.038**	-0.029*
02 Age	0.043***		-0.103***	0.151***	-0.227***	-0.289***	-0.188***	-0.085***	-0.064***	-0.062***
03 Chair	-0.004	-0.105***		0.025	0.210***	0.220***	0.034**	-0.26	0.032**	-0.012
04 Dir	0.338***	0.088***	0.035**		0.348***	0.034**	0.180***	0.105***	-0.119***	-0.130***
05 Ind_Dir	0.206***	-0.230***	0.205***	0.437***		0.916***	0.252***	-0.002	0.020	-0.016
06 Ind_ratio	0.045***	-0.310***	0.214***	0.034	0.846***		0.190***	-0.029	0.071***	0.027
07 Institutional_ratio	0.651***	-0.086***	0.025	0.223***	0.247***	0.156***		-0.046***	0.010	-0.016
08 Dir_ownership	-0.100***	-0.050***	-0.032**	0.050***	-0.034**	-0.058***	-0.189***		-0.037**	-0.085***
09 Manager_ownership	-0.094***	-0.162***	0.068***	-0.127***	0.049***	0.143***	-0.045***	-0.061***		0.091***
10 Duality	-0.131***	-0.062***	-0.012	-0.133***	-0.015	0.042***	-0.022	-0.063***	0.067***	
	01	03	06	07	11	12	13	14	15	16
Panel B: Measuring Compensation Variables										
01 Size					0.084***	0.117***	0.378***	0.672***	0.066***	-0.006
03 Chair					-0.010	-0.014	-0.029	0.005	-0.044***	-0.063***
06 Ind_ratio					0.024	0.104***	-0.009	0.115***	-0.184***	-0.225***
07 Institutional_ratio					0.198***	0.154***	0.429***	0.469***	0.103***	0.013
11 ROE	0.377***	0.007	0.064***	0.290***		0.104***	0.218***	0.128***	0.088***	0.071***
12 Tobin’s Q	0.201***	-0.012	0.057***	0.095***	0.479***		0.118***	0.083***	-0.006	-0.021
13 TotalSalary_Dir	0.619***	-0.016	0.022	0.465***	0.533***	0.162***		0.395***	0.099***	-0.012
14 TotalSalary_Manager	0.597***	0.040**	0.124***	0.531***	0.348***	0.058***	0.510***		0.097***	0.033
15 Dir_manager	0.097***	-0.044***	-0.187***	0.117***	0.090***	-0.001	0.049***	0.190***		0.878***
16 Dir_manager_ratio	-0.035**	-0.054***	-0.182***	0.031**	0.066***	-0.012	-0.084***	0.082***	0.918***	

Note

*, **, and *** represent 10% 5%, and 1% significance level, respectively.

Most companies belong to non-traditional industries, and companies established for a long time have fewer CEOs and independent directors. This indicates that when independent directors also serve as CEOs, foreign institutional investors may increase their shareholdings due to the improvement of corporate governance. In addition, whether serving as CEO and independent director is significantly negatively correlated with the year of company establishment (-0.103). Most companies established for a long time have growth potential in non-traditional industries, so there are fewer CEOs and independent directors; the proportion of foreign-funded institutions (Institutional_ratio) is significantly positively correlated with the number of CEOs and independent directors (0.034), indicating that when an independent director acts as CEO, foreign-funded enterprises may be affected by corporate governance. The proportion of independent directors (Ind_ratio) is significantly positively correlated with the salaries of directors and supervisors (Salary_Dir) and Tobin’s Q, indicating that independent directors have more board seats than directors’ and supervisors’ salaries. Employees in high-tech industries earn higher wages than in general industries. Enterprise size is positively correlated with ROE (0.084) and Tobin’s Q (0.117), and the shareholding ratio of foreign institutions is positively correlated with ROE (0.198) and Tobin’s Q (0.154) [16, 17], but with the establishment of the company showed a significant negative impact with those.

### Regression analysis

Panel A in Table 3 is the regression result measuring shareholder equity on firm performance (Y: ROE). Variable definitions are shown in Table 1. The regression equation is as follows:

$$Y_{i,t}=\alpha_{1}+\beta_{1}{Chair_{ratio}}_{i,t}+\beta_{2}{Num_{Chair}}_{i,t}+\beta_{3}{Ind_{industry}}_{i,t}+\beta_{4}{Ind_{ChainIndustry}}_{i,t}+\beta_{5}{Ind_{Tse}}_{i,t}+\beta_{6}{Education}_{i,t}+\beta_{7}{ln(Size}_{i,t})+\beta_{8}{Age}_{i,t}+\beta_{9}{Dir}_{i,t}+\beta_{10}{Ind\_ratio}_{i,t}+{\beta_{11}Institutional\_ratio}_{i,t}+\beta_{12}{Dir\_ownership}_{i,t}+\beta_{13}{Manager\_ownership}_{i,t}+\beta_{14}{Duality}_{i,t}+\beta_{15}{Dir\_manager\_ratio}_{i,t}+{Ɛ}_{i,t}$$ (1)

**Table 3:** ROE and Tobin’s Q regression analysis.

	Panel A: ROE				Panel B: Tobin’s Q			
	(1)		(2)		(1)		(2)	
Variable		VIF		VIF		VIF		VIF
Intercept	1030.9**		505.7***		0.8214		19.94**	
Chair_ratio	0.693	2.2914			0.2231	2.2923		
Num_Chair	-0.209	2.2824	0.984	6.2234	-0.073	2.2827	-0.067	6.2261
Ind_industry	1.3033	1.4358	0.2025	1.6807	0.0933	1.4365	0.137	1.6807
Ind_ChainIndustry	3.8532**	1.2042	4.0554**	1.3452	0.0927	1.2023	0.091	1.3463
Ind_Tse	-1.195	1.21	-1.1818	1.4984	-0.075	1.2104	-0.1246*	1.4978
Education	-1.089	1.1087	-0.7919	5.6676	0.0187	1.1086	-0.021	5.671
Ln(Size)	3.206***	1.6879	3.722***	1.7276	0.128***	1.688	0.119***	1.7277
Age	0.0722	1.5499	-0.0019	1.3338	-0.005**	1.5463	-0.008***	1.333
Dir	-0.031	1.7362	-0.439***	1.3161	-0.053***	1.7367	-0.015***	1.3162
Ind_ratio	-4.014	1.4447	3.5759**	1.7591	0.3627	1.4445	0.303***	1.7597
Institutional_ratio	0.0711	1.8156	0.0401**	1.6046	0.0011	1.8149	0.001	1.6039
Dir_ownership	0.1152**	1.1556	0.086***	1.0648	0.008***	1.1543	0.006***	1.0628
Manager_ownership	0.1512	1.1215	0.276***	1.0462	0.0024	1.1217	0.015***	1.0458
Duality	-0.72	1.1964	-0.9142*	1.1022	0.088	1.1963	0.0116	1.1022
Dir_manager_ratio	0.0322	1.1073	0.067***	1.2022	-0.000	1.1084	-0.001*	1.2023
Adj−R^2^	0.1145		0.2551		0.1144		0.0889	

Note

*, **, and *** represent 10% 5%, and 1% significance level, respectively.

In the past, the longer the company was established, a more complex the company structure was. Hence, the more supervisory mechanism of directors and supervisors was needed [18]. The size of the company is also positively correlated with the proportion of directors’ shareholding, indicating that more directors’ shareholding has a positive impact on ROE; it is significantly negatively correlated with the size of the board of directors and the concurrent role of the chairman as general manager. The variables representing these results are indeed consistent with previous studies and positively affect firm performance. Hypothesis 1 is supported.

In Panel B, Tobin’s Q is used as a proxy for firm performance. It is found that in the companies in group A where the CEO concurrently serves as an independent director. The positive effect still exists, but it becomes insignificant based on a company growth perspective. Further, from the perspective of corporate governance, managers hold fewer shares, which is relatively conducive to the implementation of the supervision mechanism.

Panel A in Table 4 is measured in terms of corporate costs (Y: MeanSalary_Dir). The variable definitions are the same as in Table 1, and the regression equation is as follows:

$$Y_{i,t}=\alpha_{1}+\beta_{1}{Chair_{ratio}}_{i,t}+\beta_{2}{Num_{Chair}}_{i,t}+\beta_{3}{Ind_{industry}}_{i,t}+\beta_{4}{Ind_{ChainIndustry}}_{i,t}+\beta_{5}{Ind_{Tse}}_{i,t}+\beta_{6}{Education}_{i,t}+\beta_{7}{ln(Size}_{i,t})+\beta_{8}{Age}_{i,t}+\beta_{9}{Dir}_{i,t}+\beta_{10}{Ind\_ratio}_{i,t}+{\beta_{11}Institutional\_ratio}_{i,t}+\beta_{12}{Dir\_ownership}_{i,t}\beta_{13}{Manager\_ownership}_{i,t}+\beta_{14}{Duality}_{i,t}+\beta_{15}{Dir\_manager\_ratio}_{i,t}+{Ɛ}_{i,t}$$ (2)

**Table 4:** Regression analysis: Remuneration of directors, supervisors and managers.

	Panel A: MeanSalary_Dir		Panel B: MeanSalary_Manager	
		VIF		VIF
Intercept	-10705		-124287	
Chair_ratio	50.897	4.4441	-270.13	4.4340
Ind_industry	4.6367	1.7453	245.99	1.7457
Ind_ChainIndustry	140.96	1.3086	454916	1.3070
Ind_Tse(	-105.16	1.4952	845.29	1.4959
Education	-48.317	4.4743	-102.695	4.4640
Ln (Size)	694.8***	2.0043	1129.45***	1.9950
Age	10.748***	1.2423	-27.729***	1.2460
Dir	1.3581	1.3341	37.76	1.3313
Ind_ratio	-244.39	1.7058	980.71	1.7071
Institutional_ratio	43.307***	1.8097	102.77***	1.8039
Dir_ownership	-7.858***	1.8097	-26.77***	1.0826
Manager_ownership	9.418	1.0448	8.949	1.0451
Duality	-266.78***	1.1054	-45.22	1.1040
Dir_manager_ratio	4.5004**	1.2077	2.234	1.2078
Adj−R^2^	0.2551		0.1496	

Note

*, **, and *** represent 10% 5%, and 1% significance level, respectively.

The better corporate governance, the better the regulatory effect. Based on cost considerations, the company will strictly control personnel costs. Therefore, it is measured by the remuneration of directors and supervisors. The coefficient of “Salary” is significantly positive (43.307), which means that the more foreign institutional shareholders in the company’s shares, the higher the board’s salary will be. On the contrary, the remuneration of supervisors is significantly negatively correlated (-266.78), indicating that if the management right is owned by one person, the remuneration of directors and supervisors is reduced. The reason may be that most of them are family businesses in Taiwan. Hypothesis 2 is supported.

Group B measures the average salary of managers from the perspective of company costs (Y: MeanSalary_Manager). The results are the same as those discussed in panel A. Because performance is affected by external events, the remuneration of directors and supervisors has been disclosed in an average way in recent years.

### Section 5: Conclusion

This paper studies the supervisory role of the CEO concurrently serving as an independent director on firm performance. During the study period, the annual data on CEOs concurrently serving as independent directors shows that the average number of independent directors of listed firms in Taiwan has increased from 2009 to 2019. From the perspective of firms’ costs, the remuneration for both directors and supervisors is significantly positively correlated with the proportion of institutions’ shareholding but negatively correlated with the proportion of with general manager and chairman’s remuneration.

This paper could have some management implications. How to upgrade Taiwan’s traditional industries will be a difficult problem for the sustainable development of the industry. The core of Taiwan’s next-generation industrial growth is AI. To meet the arrival of the future era of artificial intelligence and accelerate digital upgrading, the design of independent directors and related compensation will be an important issue in academic and practical research. Hence, it is appropriate that the remuneration policy varies with the economics’ circumstances.

One limitation of this work is the definition of innovation variable, it is thus suggested that future studies examine real case of innovation activity from A to Z, although such cases studies may be scarily and hardly obtained. Next, the cross-countries case studies are encouraged in this work. Finally, Big data-driven are pivotal in capital markets concerning financial institution operational efficiency [19, 20]. Through data-driven technological and process innovation capabilities, the insights about the remunerations between directors may be obtained.

## Supporting information

S1 Data (7Z)
